# Special Issue ‘Viral Infection and Autoimmune Diseases’

**DOI:** 10.3390/v14112491

**Published:** 2022-11-11

**Authors:** Hideki Nakamura, Masami Takei

**Affiliations:** Division of Hematology and Rheumatology, Department of Medicine, Nihon University School of Medicine, Tokyo 173-8610, Japan

Viral infection, which is one of the environmental factors, and human autoimmune diseases are often associated with each other. In particular, Epstein–Barr virus (EBV) and human T-cell leukemia virus type 1 (HTLV-1) are known to be involved in rheumatic diseases. However, it remains unclear how these pathogens induce rheumatic diseases and how they are involved in chronic inflammation in organs. In this Special Issue, six experts had the opportunity to extensively discuss the molecular biological mechanisms of the pathogenesis of these viruses and their involvement in clinical pathology.

These experts discuss the role of EBV, which is implicated in many autoimmune diseases. Takei et al. [1] showed that not only the EBV gene but also its related proteins were identified in rheumatoid arthritis (RA) synovium. Furthermore, they demonstrated for the first time that EBV-infected humanized mice induce RA-like bone erosion. Otsuka et al. [2] found that multiple viruses are involved in target organ and peripheral immune system abnormalities in the development of Sjögren’s syndrome (SS). In particular, they mentioned the relationship between EBV reactivation and the autoantibody production system and the importance of molecular mimicry. França et al. [3] conducted a cross-sectional study on lytic changes in EBV in rheumatic diseases in northern Brazil. As a result, it was clarified that EBV lytic changes occurred before immunosuppression by steroids in patients with systematic lupus erythematosus. Finally, Banko et al. [4] performed a meta-analysis of 79 articles on the association between RA and lymphoproliferative disorders (LPD). This analysis revealed a significant association between RA and LPD when compared with other rheumatic diseases.

Next, researchers study the involvement of HTLV-1, a virus that plays an important role in the endemic area in rheumatic diseases. Umekita [5] showed in vitro evidence for the induction of inflammation by the HTLV-1 Tax/HBZ protein, as well as clinical effects such as differences in the reactivity of biologic agents in anti-HTLV-1 antibody-positive RA. In addition, the effects of HTLV-1 on T-SPOT and the risk of HTLV-1-associated myelopathy and adult T-cell leukemia were also mentioned. Finally, Nakamura et al. [6] demonstrated the involvement of the HTLV-1 *tax* gene in SS sialadenitis in animal experiments, and mentioned the mode of transmission of HTLV-1 to salivary gland epithelial cells and the inflammation-initiating mechanism. We also suggested that HTLV-1 suppresses autoantibody production by directly affecting follicular dendritic cells.

Based on the results of the four review articles, one brief article, and one meta-analysis, further research on the involvement of viral infections in rheumatic diseases is necessary, and further development is highly expected in the future. Through this Special Issue, we believe we have gained a better understanding of how viruses are activated and affect lymphoid and epithelial cells. However, many basic and clinical questions remain about the transition from the activation of the innate immune system to the acquired immune system. As guest editors, we would like to thank the reviewers for their professional comments based on their historical perspectives and current knowledge, despite their busy schedules. We would especially like to thank Louies and other members of the *Viruses* editorial office for their punctual and strong support. Finally, we would like to express our sincere gratitude to all authors for their significant medical and scientific contributions.
